# Multidisciplinary Approach to Suspected Sudden Death Caused by Arteriovenous Malformation Rupture: A Case Report

**DOI:** 10.3390/medicina57070644

**Published:** 2021-06-23

**Authors:** Federico Giuseppe Patanè, Massimiliano Esposito, Andrea Giovanni Musumeci, Monica Palermo, Marco Torrisi, Monica Salerno, Angelo Montana

**Affiliations:** 1 Legal Medicine, Department of Medical, Surgical and Advanced Technologies, “G.F. Ingrassia”, University of Catania, 95123 Catania, Italy; federicopatane90@gmail.com (F.G.P.); marcotorrisi1992@gmail.com (M.T.); monica.salerno@unict.it (M.S.); angelomontana49@gmail.com (A.M.); 2 Radiology Unit 1, Department of Medical Surgical Sciences and Advanced Technologies “G.F. Ingrassia”, University-Hospital Policlinico, University of Catania, 95123 Catania, Italy; andreagiovannimusumeci@gmail.com (A.G.M.); monica.palermo91@gmail.com (M.P.)

**Keywords:** arteriovenous malformations, sudden death, pathological observations

## Abstract

Arteriovenous malformations (AVMs) are rare congenital conditions with a prevalence of less than 1% and are mostly asymptomatic. However, these malformations can suddenly cause intense pain or bleeding, leading to life-threatening medical problems. This report presents a case of an unexpected death in a 37-year-old previously healthy woman due to an intra-cerebellum arteriovenous malformation rupture identified during autopsy. While infective processes where preliminarily excluded, a Post Mortem Computed Tomography (PMCT) identified a tetra ventricular hemorrhage and intra-cerebellum hemorrhage. Toxicological examination was negative for most substances of abuse. During autopsy an intense hemorrhagic infiltrate in the subarachnoid space was observed. After formalin fixation of the brain the cerebellum showed hemorrhagic infarction on fourth ventricle sides, as well as several small reddish infarctions across the entire cerebellum parenchyma. Histological examination of the brain and cerebellum showed a suffusion of erythrocytes in the sub-arachnoid region. Evidence of an arterio-venous malformation, with several intertwine vessels of variable diameter, surrounded by hemorrhagic evidence. The autopsy played a crucial role in identifying the location and the possibly affected vessel, as well as defining the cause of death. It is necessary to have a greater number of autopsies to make an epidemiological contribution. Furthermore, it is crucial to create a multicenter data network with other authors from other departments to improve information about epidemiological, clinical, diagnostic and therapeutic data. Most brain AVMs as cause of death are often undiscovered.

## 1. Introduction

The most common cause of intracerebral haemorrhage in young people is due to vascular malformations. The risk of bleeding from a vascular malformation is estimated to be 2% per year in cases of arterio-venous malformation (AVM) [1,2,3,4]. Mortality is 10–15% of cases in cerebral haemorrhages caused by AVM [5].

Congenital disorders are conditions present at birth regardless of their cause. Several diseases are caused by congenital disorders, but many disorders are asymptomatic for the rest of a person’s life. In some cases, these disorders can be identified only accidentally or after death. An AVM is an example of such a disorder. In a recent review, the authors identified a prevalence of 0.94–1.21 cases per 100 × 10^3^ person-years, thus arteriovenous malformations are rare congenital disorders [6,7,8].

Most lesions present suddenly with cerebral hemorrhage (reported frequencies from 30% to 82% of cases) [9,10]. Treatment is primarily aimed at prophylaxis against new spontaneous intracranial hemorrhage [11,12], and it usually includes procedures like endovascular embolization, radiosurgery, or microsurgical resection.

We present a case report of AVM related-death in order to improve our overall knowledge about its complications and life-threating consequences. A full autopsy, between radiological results and histological presentation, could give a complete overview of such malformations.

## 2. Case Report

The forensic pathologist was called to examine a death of a 37-year-old woman, found death in the morning, without evident signs of violence; no information was provided about the previous evening. This study was in accordance with the 1964 Declaration of Helsinki or the institution’s ethical standards, subject to informed consent obtained from relatives.

### 2.1. Circumstantial Data

The young woman was previously healthy and of normal weight. All available court files were analyzed. The woman was in did not smoke, and did not drink alcohol. The general practitioner was consulted, too, and reported that her blood pressure was normal, and she was not taking any medications, and she never suffered from migraines in the past. The previous evening, she had spent some time with one of her friends. During the evening she suffered a sudden headache, nausea and unstoppable vomiting. Because of the onset of such symptoms, she returned home earlier than intended and lay on her bed to rest for the night; she apparently died during the night. No other symptoms were previously reported she was found death by her son during the next morning. Emergency medical doctors confirmed death status and reached law enforcement authorities in the context of a sudden unexpected death at domicile. The local prosecutor called the forensic pathologist to investigate the crime scene and to perform an autopsy to exclude any murder intention from others.

### 2.2. Crime Scene Investigation

The dead body was located on the third floor of a condominium. The apartment was in order and clean. The body was still lying on the bed with blankets partially removed. On the night table, located at the right of the bed, the investigators found a roll of paper. A plastic tray was lying on the floor near the bed containing some dried brownish liquid. Under the head, the pillow and the sheet were stained with dried red liquid. Red and white liquid came out from her mouth.

The woman wore a t-shirt, hoodie and trousers. The upper clothes were torn apart frontally, due to cardiac resuscitation efforts. The body temperature was 38.8 °C, while the ambient temperature was 18.7 °C. Lividity was fixed, consistent with her body position. Rigor mortis was in the process of formation. Only minor scars and tattoos were found on her body, initially excluding any violence suffered during her last days. The post-mortem interval (PMI) was estimated between 4 and 8 h before the cadaveric inspection.

### 2.3. Post-Mortem Computed Tomography (PMCT)

**Figure 1:**
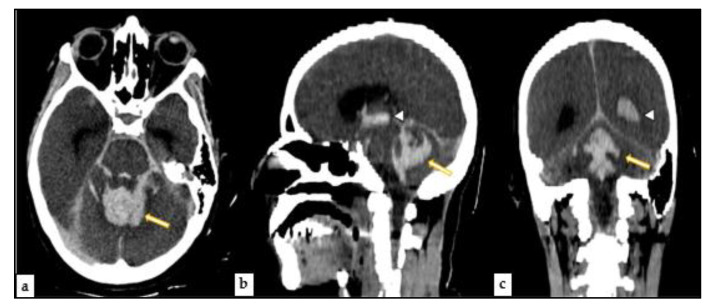
PMCT—(**a**) Axial image revealed signs of intracerebral hemorrhage of the cerebellum (yellow arrow). (**b**) Sagittal view showed another hemorrhage site, the basal ganglia (white and yellow arrows). (**c**) Coronal view of the brain offered another type of hemorrhage view (white and yellow arrows).

After the approval of the public prosecutor to carry out this examination, the corpe was transported to the CT room next to the autopsy room, and was subjected to a CT investigation. The analysis of the CT images was conducted by specialist forensic radiologists. The corpse was placed inside two sterile and waterproof bags to avoid contamination of the equipment. Scans were performed in the supine position from head to toe. Post-mortem imaging was performed using a helical 16-slice CT scanner (Philips Ct Brillance 16, Catania, Italy) The time interval between death and PMCT was about 48 h, PMCT showed multiple signs of intracerebral hemorrhage, originating from the cerebellum and basal ganglia (Figure 1).

**Figure 2:**
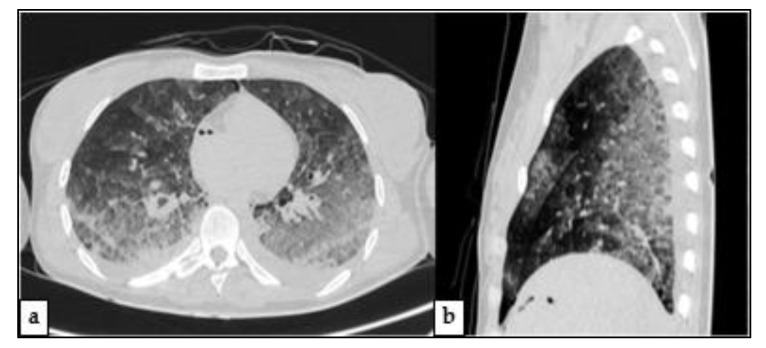
–PMCT of Chest—(**a**) Axial image revealed high attenuation areas, mainly ground glass, with a gradient appearance (in dependent regions). (**b**) Sagittal view of chest shows that almost only dorsal aspects of lungs are affected by opacity alterations.

**Figure 3:**
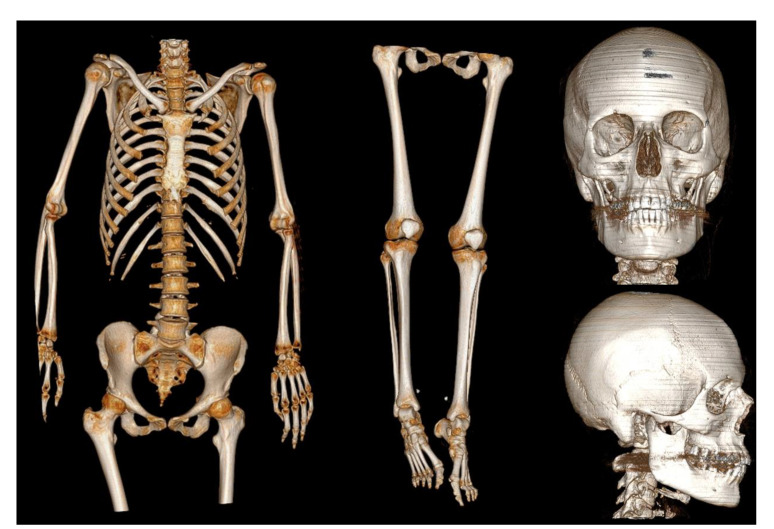
–3D reconstructions of PMCT, showing no evident signs of trauma and fractures (from the left: upper body, lower limbs, frontal view of skull, lateral view of skull).

In the chest region, some zones of grater opacity mostly of ground glass type, confined in dependent regions (Figure 2), explainable as normal distribution of blood and air volume after death [13]. No fractures were identified on total body PMCT (Figure 3).

### 2.4. Autopsy

Autopsy was performed about 48 h after death: external examination was unremarkable.

#### 2.4.1. Technique

**Figure 4:**
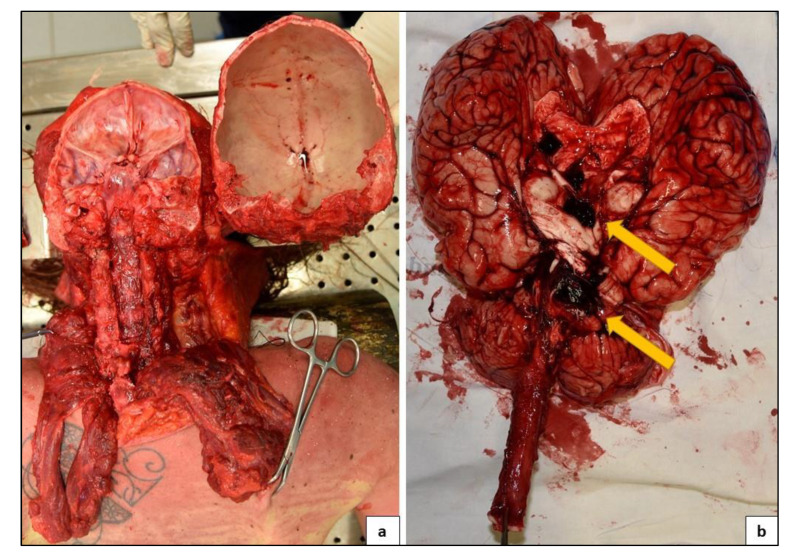
Result of removal of the brain, cerebellum, with the posterior access. Preserved integrity of the vascular structures (**a**). Arrows reveal the site of the hemorrhage (**b**).

The examination of the head and neck was performed through a posterior approach, this cut allowed to respect cervical medulla, pons, cerebellum and brain integrity (Figure 4). Vertebral arteries were extracted and preserved along with the carotids and the circle of Willis. A section of the optic nerves was performed at the chiasm. The section continued dissecting the tentorium, preserving the brain and cerebellum. The brain had herniated signs to the opposite side of the bleeding. During the autopsy, the larynx and trachea were open, and there was blood and mucus inside. Autopsy was completed by dissecting the remaining anatomical regions after a thoracic-abdominal section, followed by organs extraction taking care to preserve anatomical relations as much as possible. All organ samples were preserved in formaldehyde and stained in Hematoxylin Eosin for histological analysis. All cephalic, thoracic and abdominal tissues were then examinated.

#### 2.4.2. Central Nervous System Findings

**Figure 5:**
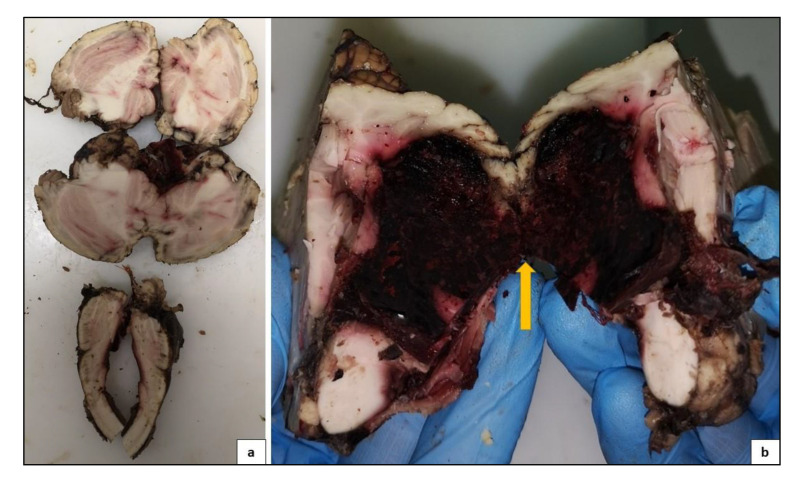
Different cutting surfaces on the cerebellum and oblongata medulla. (**a**) First cutting surface performed along the median axis of the oblongata medulla and across the cerebellum showing hemorrhagic infarction on fourth ventricle sides, as well as several small reddish infarctions across the entire cerebellum parenchyma. (**b**) Second cutting surface on the superior part of the cerebellum shows a massive hemorrhagic infarction that extends to the edge of the fourth ventricle.

PMCT findings were confirmed: a massive area of hemorrhagic suffusion was identified after dissecting the cerebellum, mainly in the anterior inferior cerebellar artery perfusion area. Moreover, on the medial side of the right cerebellar hemisphere, a protrusion region, with a range of less than 2 cm, was identified. The exact affected part of the anterior inferior cerebellar artery was not identified. An intense hemorrhagic infiltrate in the subarachnoid space was observed. Both neurological and vascular structures were examined during autopsy and after formalin fixing. After coronal section, the brain was regular in size and displayed small diffuse cerebral hemorrhages of the right frontal and parietal lobe. The right side of the brain stem showed the same lesions (Figure 5).

#### 2.4.3. Other Findings

The heart was 12 × 10.5 × 5 cm and weighed 350 g. The lungs were of regular shape and volume, measuring 23 × 16 × 3.5 the left and 23 × 16 × 6 the right. The left lung weighed 950 g and the right one 750 g, a small white foam protruding from the lung parenchyma. Other organs showed only an intense vascular congestion.

A toxicological analysis was performed and excluded substances of abuse in the liquids and organs of the cadavers.

### 2.5. Histological Findings

**Figure 6:**
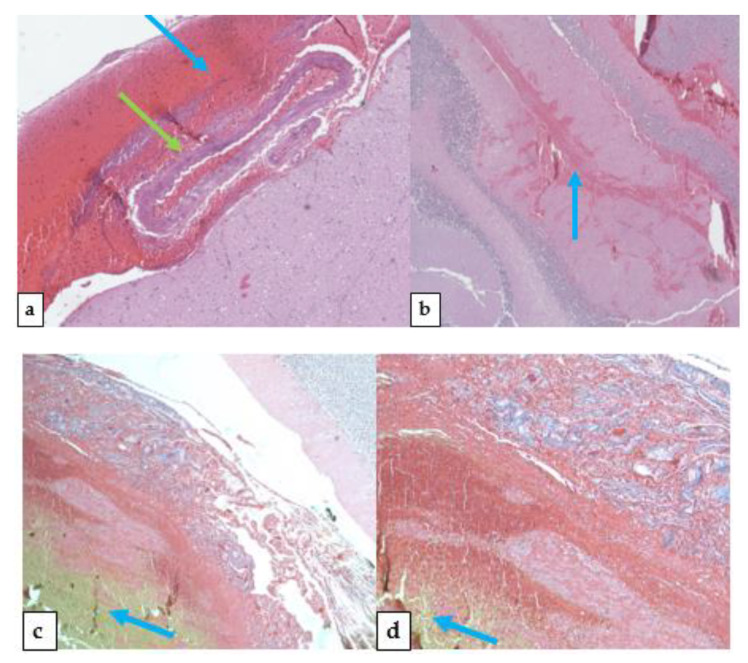
Different histological findings across the cerebellum. Peripheral examination showed flattened vessels green arrow) ((**a**)-green arrow indicates flattened vessels, blue arrow indicates the points of massive bleeding) and massive hemorrhage (**b**). Massive hemorrhage signs were also found inside the cerebellum parenchyma (**b**). During sampling procedures, the specific broken vessel was not identified, but the overall vessel structure was more represented than usual, especially on the upper side of the cerebellum, confirming the underlying arterio-venous disorder. Masson’s trichrome stain of the cerebellum (**c**,**d**): cytoplasm, keratin, acidophilic granulocytes in red; collagen in blue; erythrocytes in yellow.

Microscopy with hematoxylin-eosin staining was carried out and in the brain cortex samples, it was possible to identify a suffusion of erythrocytes in the sub-arachnoid region. In cerebellum samples it was possible to identify a massive suffusion of erythrocytes and other blood elements along the margin of the structures. Most arterial and venous vessels were crumpled with a wavy endothelium. Vascular structures near the cerebellum vermis showed evidence of an arterio-venous malformation, with several intertwine vessels of variable diameter, surrounded by hemorrhagic evidences. In some regions, the hemorrhagic suffusion also affected the cerebellum parenchyma. A Masson’s trichrome stain was performed, which confirmed the AVM. (Figure 6). The rest of the samples (heart, lungs, liver, kidneys, spleen) were not significant, however. They did show intense vascular congestion.

### 2.6. Cause of Death

The cause of death was due to a massive intracerebral hemorrhage caused by a ruptured AVM located in the choroid plexus of the fourth ventricle. The compressive action caused by the cerebral hypertension due to the hemorrhage caused an impairment of vital functions. The symptoms she suffered the previous evening, along with PMCT, autopsy and histological findings, support this diagnosis.

## 3. Discussion

**Figure 7:**
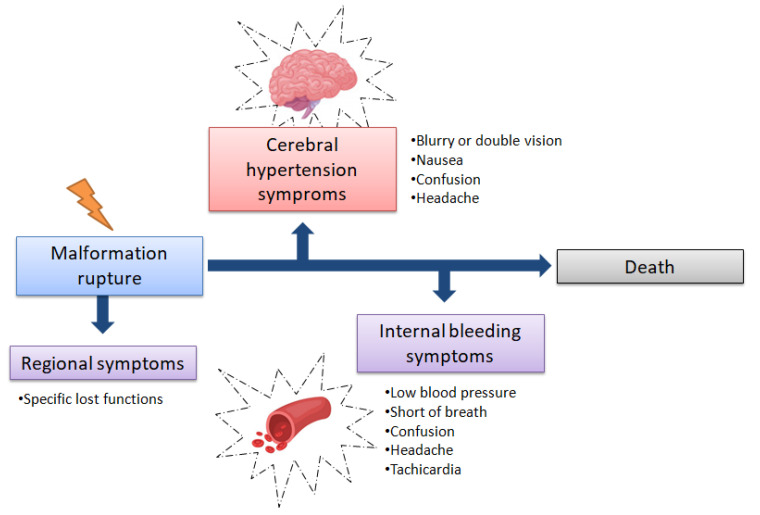
As time passes, the first symptoms of a vessel malformation rupturing in the brain become less and less specific, passing from regional symptoms (based on malformation rupture location), to a more generic hypertension and bleeding symptoms. In such conditions the healthcare professional should view the symptoms as a whole, performing specific diagnostic procedures as soon as possible to identify the localization of the rupture.

In the situation where patients, without clear signs and symptoms, access an emergency department, a diagnosis could be a complex task. However, when the patients themselves do not recognize their symptoms as an alarm for a pathological condition, the consequences could be very risky. Moreover, an underlying condition of a congenital disorder such as AVM, could lead this missed self-recognize symptom to a life-threatening condition, or even death (Figure 7).

This report highlights a death that respected the World Health Organization (WHO) criteria of sudden death definition [14], which happened in a domestic context and caused by an AVM rupture.

It is useful to highlight that arteriovenous malformations are rare congenital disorders, where their rupture could threaten the life of the patient. Their rupture is even rarer, and usually happens during the course of life with a peak occurring in patients 20–40 years old. Only few cases of this rare condition have been reported in pediatric patients [15].

Many studies were made about such malformation ruptures, especially during pregnancy [16,17,18], treatments [19,20], or after repair [21]. Treatment of such malformations is contradictory.

Unlike clinical and therapeutic advances, the literature on sudden death due to AVMs is poor. In fact, a retrospective analysis, conducted by Matschke et al., of 5432 autopsies performed by the Institute of Neuropathology, publicized only 3 cases of sudden death caused by hemorrhage of AVMs [22]. All deaths occurred in asymptomatic patients and in two cases it happened outside the hospital. The same authors performed a literature review about death by AVM and they concluded that they were exceedingly rare: a search of the period from 1966 to 2004 yielded only 7 cases. Karhunen et al. [23] reported only five cases in 8000 consecutive autopsies of suspected AVM death. Rosen and Azzopardi presented a total of five sudden deaths in children from cerebellar AVM [24,25].

Racette, S. et al. [26] published a case report of a 14-year-old girl who was found dead on her bed. She suffered only for mild asthma. After school, she started to feel sick with nausea, headache and vomit. The next day she was found dead. A complete autopsy was performed and a cerebral hemorrhage was observed. Microscopically, the lesion showed different caliber, thick-walled, vascular channels, gliosis, hemosiderin deposits, abnormal vessels in the subarachnoid space. Thus, forensic pathologists may supply a crucial contribution on epidemiologic data on AVMs, above all, if autopsies were made in all cases of sudden death in young people without a history of longstanding hypertension.

A particular case report showed a sudden death in a kid, caused by a broken vascular malformation with an uncommon site, not recognized macroscopically but only on histological investigation [27]. In this case, an MPCT scan did not show the AVM, highlighting the crucial role of autopsy in this kind of death.

Efawal, M. A. et al. [28] published a particular case report about an AVM death preceded by blunt trauma. In this case, one must consider if the rupture was coincidental or a direct result of the trauma, in that specific case a head injury caused by a punch to the face. Finally, with the crucial help of autopsy, the verdict was that of manslaughter. Determining the cause of death can be complex when side effects of treatments or drug abuse could be a determinant factor in the death [29,30].

The present case shows that AVMs are a possible cause of sudden and unpredicted death, and forensic pathologists should be able to recognize this injury and make a differential diagnosis from the other types of unexpected death. It is necessary to increase the number of autopsies to make an epidemiological contribution.

Furthermore, as in other rare diseases, secondary deaths to AVM are important to record for epidemiological reasons, but their scarcity means that such data in a multicentre network database may not have a huge impact on public health. Most brain AVMs as cause of death are often unidentified [31]. It is crucial to create a multicenter and multispecialist data network for the adequate interdisciplinary diagnosis in autopsy cases, in order to improve information about epidemiological, clinical, diagnostic and therapeutic data. It would be an important benefit first for single cases and then for the community, as it would give more reliable data.

## 4. Conclusions

In the present article, we report a rare case of sudden death caused by a rupture from an AVM that was not shown during macroscopic examination but only due to histological examination. PMCT scans excluded trauma and massive abdominal lesions; although it was impossible to recognize an AVM, mainly because the lack of contrast medium administration, the radiologist correctly identified intracerebral hemorrhage and precisely located the affected area. PMCT helped the pathologist in establishing the cause of death, leading to an accurate examination of the brain. It was a useful tool, which should be applied to the routine autopsy protocol [32,33,34]. Autopsy plays a crucial role in identifying the cause of death beyond any doubt [35,36,37,38,39]. Many unexpected deaths are currently associated with cardiovascular diseases and in many cases without any type of confirmation. Judiciary autopsies are performed only if needed, as in this case report; hospital autopsies in unexpected deaths should be performed to know exactly the incidence of rare disorders, such as AVMs. We still have much more to learn from the dead.

## Data Availability

All data are included in the main text.
